# *Fasciola hepatica*: variations in redial development and cercarial production in relation to the geographic origin of the parasite

**DOI:** 10.1051/parasite/2013034

**Published:** 2013-09-24

**Authors:** Daniel Rondelaud, Rodrigo Sanabria, Philippe Vignoles, Gilles Dreyfuss, Jorge Romero

**Affiliations:** 1 INSERM U 1094, Faculties of Medicine and Pharmacy 87025 Limoges France; 2 CEDIVE, Fac. Cs. Veterinarias, Universidad Nacional de La Plata Alvear 803 7130 Chascoműs, Buenos Aires Argentina

**Keywords:** Argentina, Cercaria, *Fasciola hepatica*, France, *Galba truncatula*, Miracidium, Redia

## Abstract

Two hundred *Galba truncatula*, originating from a French population, were subjected to single-miracidium infections with an allopatric isolate (origin, Argentina) of *Fasciola hepatica.* The control group was constituted by 100 snails coming from the same population and exposed to sympatric miracidia of *F. hepatica* according to the same protocol. Snail samples were collected bimonthly from both groups between day 14 and day 112 p.e. (at 20 °C) and snail dissections were performed to count free rediae, intraredial morulae and free cercariae. Third and fourth generation rediae were significantly more numerous in the allopatric group, while the number of first generation rediae was significantly lower. In the sympatric group, the decrease in the number of intraredial morulae in the first, second and third redial generations was significantly faster. Free cercariae within the snail body were significantly more numerous in the sympatric than in the allopatric groups, whatever the date of snail dissection. The changes in redial development and cercarial production noted in the Argentinean group might be due to the evolution of South American flukes in a divergent way after the introduction of foreign infected ruminants in this continent from the 15th century.

## Abstract (fr)

Deux cents *Galba truncatula*, provenant d’une population française, ont été soumis à des infestations monomiracidiennes individuelles avec un isolat allopatrique (origine Argentine) de *Fasciola hepatica.* Le groupe témoin est constitué par 100 limnées issues de la même population et exposées à des miracidiums sympatriques de *F. hepatica* selon le même protocole. Des échantillons de limnées ont été prélevés tous les 15 jours dans les deux groupes entre le 14^e^ et le 112^e^ jour (à 20 °C) et des dissections ont été réalisées pour compter les rédies indépendantes, les morulas intra-rédiennes et les cercaires libres. Les rédies de troisième et de quatrième génération sont significativement plus nombreuses dans le groupe allopatrique alors que le nombre de rédies de première génération est significativement plus faible. Dans le groupe sympatrique, le nombre de morulas intra-rédiennes diminue plus rapidement dans la première, la deuxième et la troisième génération. Les cercaires libres dans le corps du mollusque sont significativement plus nombreuses dans le groupe sympatrique que dans le groupe allopatrique, quelle que soit la date de la dissection. Les changements dans le développement rédien et la production cercarienne, que l’on note dans le groupe argentin, pourraient être dus à l’évolution des douves sud-américaines dans une voie divergente après l’introduction de ruminants infestés d’origine étrangère sur ce continent à partir du 15^e^ siècle.

## Introduction

Three and sometimes four redial generations of *Fasciola hepatica* develop one after another in the body of the snail host [12, 13]. Leaving aside the first redia of the first generation which only produces daughter rediae, each generation first produces daughter rediae and secondly cercariae. These last larvae become free later within the snail body and, after a period of several days during which they accumulate glycogen and fatty acids, finally exit from the snail to encyst on an aquatic plant and turn into metacercariae [6]. In Central France, a production ranging from a mean of 94–220 cercariae of *F. hepatica* was noted when the method by Rondelaud et al. [11] was used to raise 10 populations of *Galba truncatula* experimentally infected with two local miracidia per snail [14]. According to Rondelaud et al. [12], the number of free rediae of *F. hepatica* in sympatric bimiracidial infections of *G. truncatula* ranged from 19 to 25 per snail.

This cercarial production of *F. hepatica* sometimes differs when allopatric miracidia are used for experimental infections. A mean production of 651 and 897 metacercariae was reported by Sanabria et al. [15, 16] for two populations of French *G. truncatula* (out of the 10 cited above) when snails were infected with an Argentinean isolate of miracidia (2 per snail). In contrast, the numbers of metacercariae were significantly lower (a mean of 166 and 222, respectively) when the same snail populations were infected with a French miracidial isolate according to the same protocol [15, 16]. Even if the patent periods were significantly longer in snails infected with the Argentinean isolate, the numbers of free rediae counted in Argentinean and French groups were close to each other [16]. In view of these findings, it is difficult to understand why there was 3.9–4.0 times more metacercariae in snail groups infected with the Argentinean isolate, whereas redial burdens were similar in the Argentinean and French groups. As this enhanced production of *F. hepatica* cercariae in French snails infected with the Argentinean isolate was very interesting for commercial production of metacercariae [14], it was useful to study the development and production of *F. hepatica* redial generations in sympatric and allopatric infections of *G. truncatula* via the following two questions: does the number of free rediae in each generation change when French snails are infected with Argentinean miracidia? Is the dynamics of cercarial differentiation within these rediae affected by the geographic origin of the miracidia? To answer these questions, experimental infections of French *G. truncatula* with an Argentinean isolate of miracidia were carried out to count free rediae, intraredial morulae and free cercariae in snails dissected at regular intervals. Controls were constituted by the same population of *G. truncatula* infected with a French isolate of miracidia and raised according to the same protocol.

## Materials and methods

### Snails and parasites

The population of *G. truncatula* was living in a road ditch (46°40′27″ N, 1°21′21″ E) along a meadow on the commune of Chitray, Department of Indre (Central France). These snails were known to be highly susceptible to local *F. hepatica* [4, 14] and the upper shell height of adults ranged from 11 to 12 mm. Three hundred snails, measuring 4 mm in height, were used for this experiment. Eggs of the Argentinean isolate of *F. hepatica* were collected from cattle gall bladders at Buenos Aires while those of the French isolate came from the gall bladders of cattle slaughtered at Limoges (Central France). Both types of eggs were washed several times with spring water and were incubated for 20 days at 20 °C in the dark [9].

### Experimental protocol

Two hundred snails were exposed to the Argentinean isolate of *F. hepatica* (allopatric group) while the control group was composed of 100 *G. truncatula* subjected to French miracidia (sympatric group). Each snail was exposed to a single miracidium for 4 h at 20 °C in 3.5 mL of spring water. Snails were then raised in groups of 10 individuals in 14 cm Petri dishes (volume of spring water, 60 mL) for 30 days according to the method by Rondelaud et al. [11]. Food consisted of dried lettuce leaves and dead grass (*Molinia caerulea*) leaves, while several stems of live *Fontinalis sp.* ensured oxygenation of the water layer. The dissolved calcium in spring water was 35 mg/L. Petri dishes were placed at a constant temperature of 20 °C (±1 °C) and natural photoperiod of 10 h light. A daily surveillance was made to change spring water and food if necessary.

Samples of 10–25 snails (Argentinean group) or 5–10 snails (French group) each were collected every two weeks from day 14 post-exposure (p.e.) to day 112 and from day 14 to day 84, respectively. This two-week interval was chosen in order to have a sufficient number of infected snails for each sampling date. No surviving snails were found after day 112 (Argentinean group) or day 84 (French group). After their collection, snails were dissected under a stereomicroscope to determine the type of redial development (normal or abnormal: [12, 13]) in relation to the life or the death of the first mother redia (the development of redial generations is normal when the first mother redia did not die during the first or the second week of infection). Only snails showing a normal development of redial generations were considered in the present study. Free rediae containing germinal masses were counted according to their generation. Each redial generation was identified using pharynx morphology and time of infection [12, 13]. Empty rediae were not counted in this study. The body wall of each free redia was then opened using a thin scalpel blade to remove and count rounded morulae. Free cercariae within the snail body were also counted. In contrast, metacercariae present in Petri dishes from day 42 p.e. before snail dissection were not counted in the present study.

### Parameters studied

Prevalence of *F. hepatica* infection was established by calculating the ratio between the number of snails containing an active infection and that of snails dissected. Another parameter was the growth of infected snails between miracidial exposure and day 84 p.e. For each redial generation, the number of free rediae containing germinal masses and the quantity of morulae were considered. The last parameter was the number of free cercariae within the snail body. Individual values recorded for the shell heights of infected snails and the numbers of rediae, morulae or free cercariae were averaged and their standard deviations were calculated considering the snail groups. A χ^2^ test and one-way analysis of variance were used to establish levels of significance. All the analyses were made using the Statview 5.0 software (SAS Institute Inc., Cary, NC, USA).

## Results

### Main characteristics of infection

In the Argentinean group, 167 snails were dissected up to day 112 and 42 contained an active infection (prevalence, 25.1%). Sixty-one snails were dissected in the French group up to day 84 and 38 snails were infected (prevalence, 62.2%). A significant difference (χ^2^ = 27.07, *p* < 0.001) between these values was noted. Among these infected snails, 41 (Argentinean group) and 35 (French group) showed a normal development of redial generations, while the others (1 and 3, respectively) had an abnormal development. The growth of infected snails from miracidial exposure up to day 84 p.e. was 2.1 ± 0.3 mm in the Argentinean group and 1.9 ± 0.2 mm in the French group. The difference between these values was nonsignificant.

### Number of free rediae for each generation

**Figure 1.**
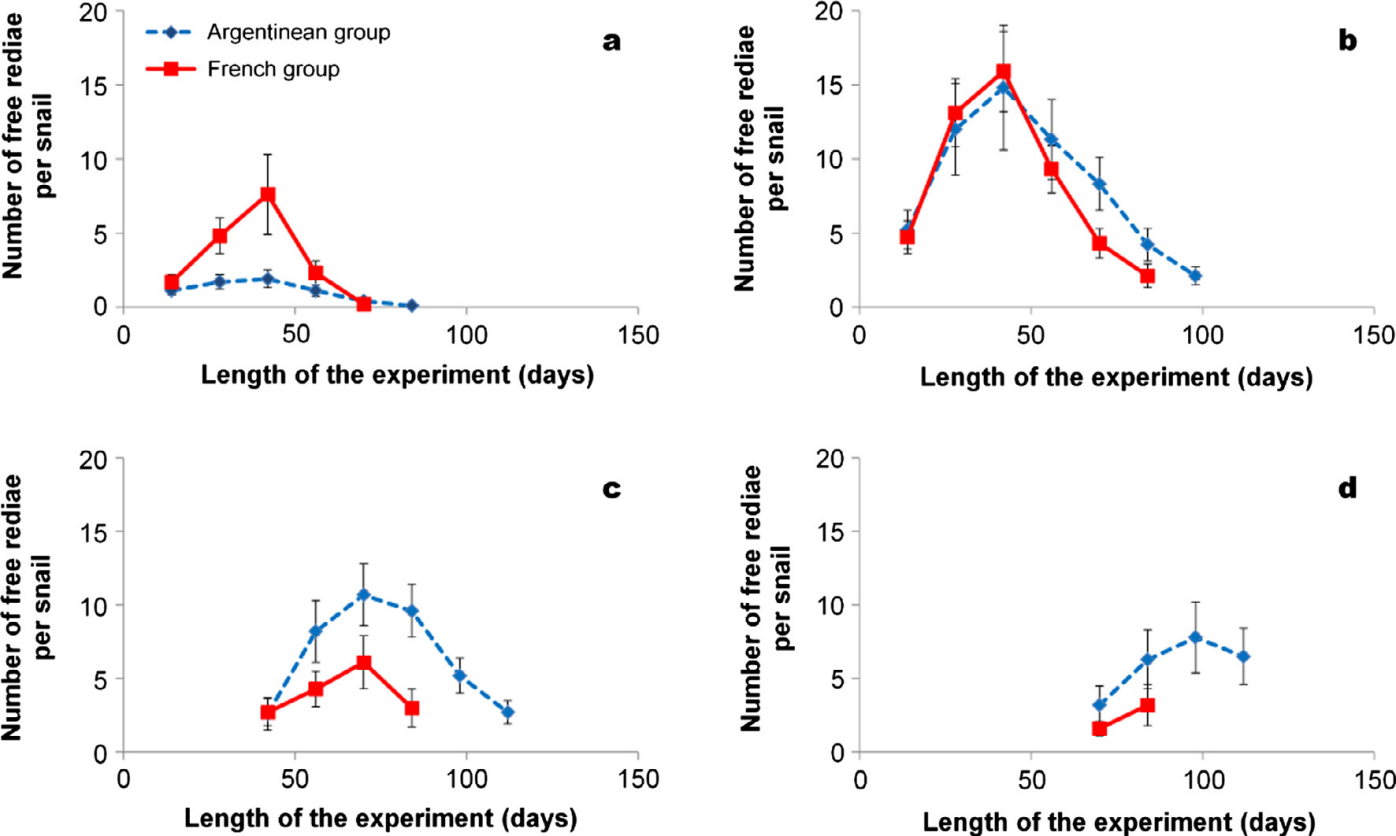
Mean numbers (standard deviation) for *F. hepatica* free rediae per infected snail in two groups of *G. truncatula* in relation to their generation: first generation other than the first mother redia (1a), second generation (1b), third generation (1c) and fourth generation (1d).

In both groups of snails (Figure 1a), the number of first generation rediae (other than the first mother redia) peaked at day 42 p.e. and decreased thereafter up to day 70 (French group) or day 84 (Argentinean group). However, the number of these rediae at day 42 was significantly lower (*F* = 19.63, *p* < 0.001) in the Argentinean group than in the other snails. A similar development was also noted for second generation rediae (Figure 1b) with peaks at day 42 and a subsequent decrease of numbers until day 84 (French group) or day 98 (Argentinean group). The difference between the numbers of these rediae at day 42 was nonsignificant. The first free rediae of third generation (Figure 1c) were observed from day 42 in both groups. Their numbers peaked at day 70 and decreased thereafter up to the end of experiment. At day 70, these rediae were significantly (*F* = 12.18, *p* < 0.01) more numerous in the Argentinean group. A similar finding was also noted for fourth generation rediae (Figure 1d). These rediae appeared at day 70 and their number at day 84 was significantly greater (*F* = 7.56, *p* < 0.05) in the Argentinean group than in the other snails.

### Redial production

**Figure 2.**
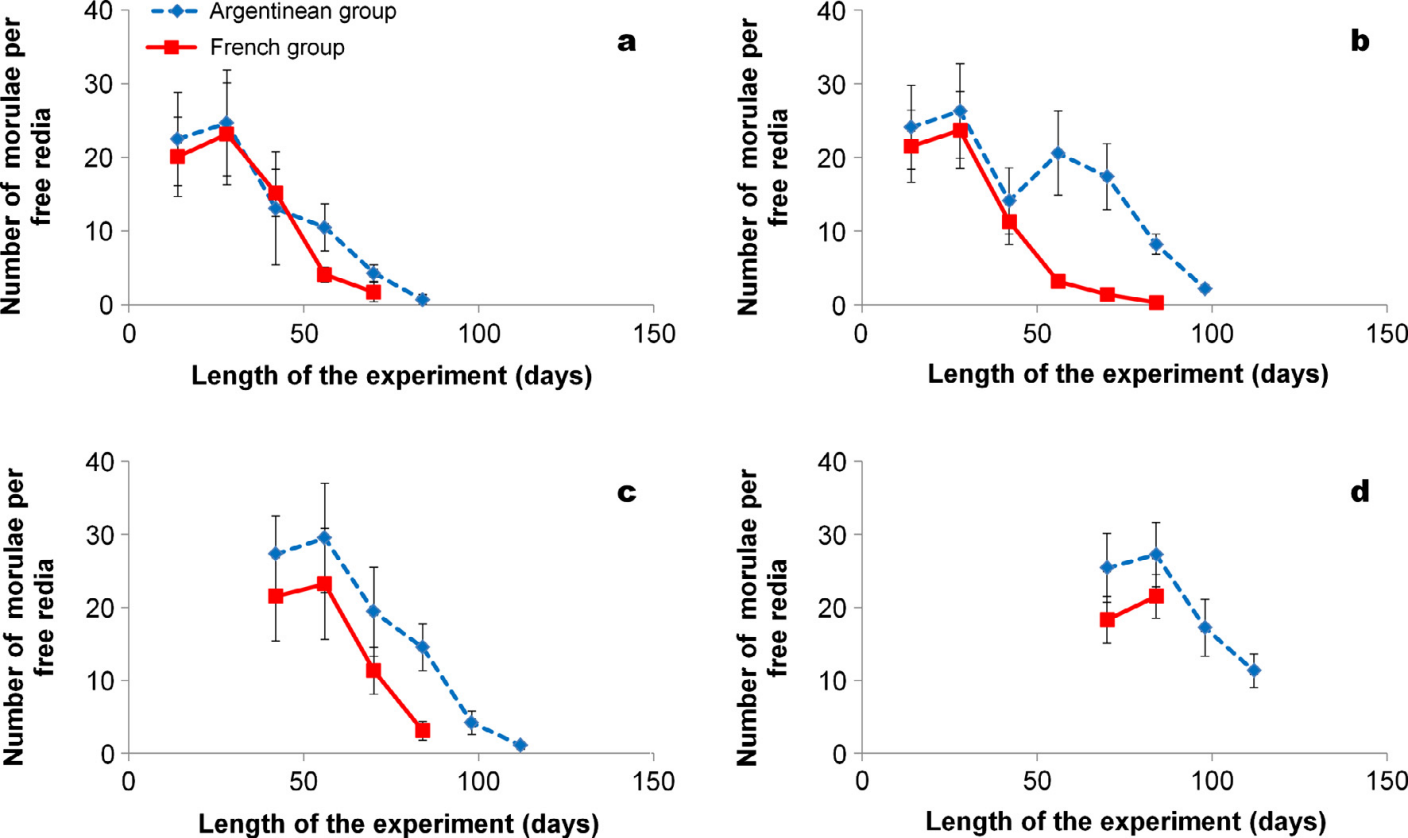
Mean numbers (standard deviation) for *F. hepatica* morulae per free redia in two groups of *G. truncatula* in relation to the redial generation: first generation other than the first mother redia (2a), second generation (2b), third generation (2c) and fourth generation (2d). No surviving snails were noted in the French group after day 84.

In the four redial generations (Figure 2), the numbers of morulae decreased with increasing length of experiment. However, this decrease in each generation was faster for the French group than in the Argentinean one. For example, second generation rediae in the Argentinean group still contained a mean of 17.4 morulae at day 70 (Figure 2b), while there was a mean of 1.4 morulae in each corresponding redia of the French group. Significant differences in the numbers of morulae between the Argentinean and French groups were noted after day 42 in the first (*F* = 17.05, *p* < 0.001), second (*F* = 43.80, *p* < 0.001) and third (*F* = 51.19, *p* < 0.001) redial generations.

**Figure 3.**
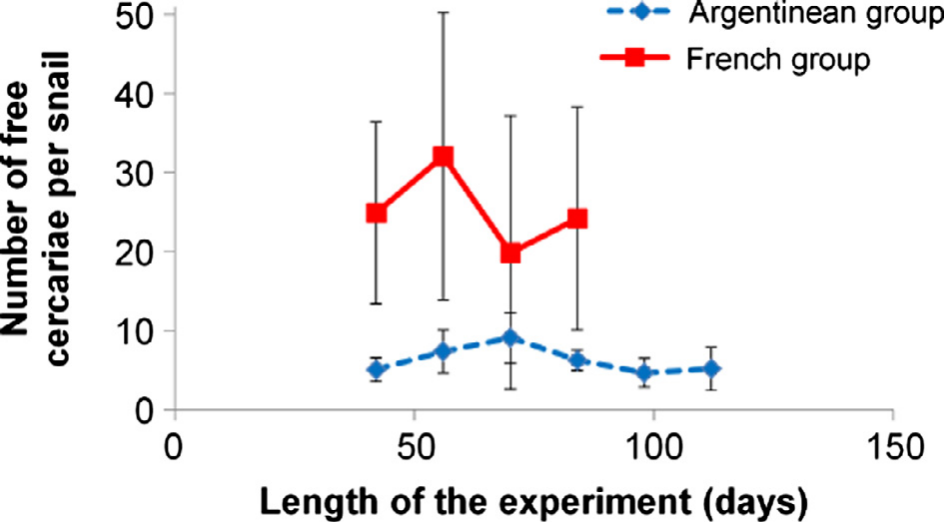
Mean numbers (standard deviation) for *F. hepatica* free cercariae per infected snail in two groups of *G. truncatula*. No surviving snails were noted in the French group after day 84.

The numbers of free cercariae counted in the body of infected snails after their dissection are given in Figure 3. These cercariae were significantly (*F* = 80.78, *p* < 0.001) more numerous in the French group between day 42 and day 84, whatever the date of snail dissection. In the Argentinean group, less cercariae were recorded.

## Discussion

Compared to the developmental pattern of redial generations observed in the French group, several differences were noted in snails infected with Argentinean miracidia. First, the rediae of first generation other than the first mother redia were limited in number in the last group. Secondly, the rediae of third and fourth generations were more numerous. As the growth of infected snails up to day 84 p.e. and the number of second generation rediae were similar in both snail groups, the most valid explanation for these changes in the Argentinean group is to relate them to the volume of the snail host. According to Rondelaud et al. [12], second generation rediae produced by the first mother redia became free during the same period than the other first generation rediae which emerged later from the sporocyst. The lower number of first generation rediae in the Argentinean group and, as a consequence, the greater space available in the body of snails during this period would be at the origin of the formation of a greater number of third and fourth generation rediae. An argument supporting this explanation is the relationship between the number of rediae developing in a snail and the size of this snail host, as previously reported by Zischke [17] and Rondelaud and Barthe [10] for *Echinostoma revolutum* and *F. hepatica*, respectively.

Even when the number of intraredial morulae in each redial generation was close to each other in both groups of snails, the numerical decrease of these germinal masses was faster in the French group, whatever the redial generation. This finding suggests that morula production in each redial generation of the Argentinean group would be higher and would last longer over time. Despite the slight decrease in morula production noted at day 42 in second generation rediae of the Argentinean group (Figure 2b), the other generations did not show such decrease, so that our results did not allow to conclude to the presence of a second morula growth when rediae still contained long-tailed cercariae. In our opinion, the formation of intraredial morulae in the Argentinean group would be a continuous process over time up to the decrease and disappearance of these germinal masses. This hypothesis is supported by the presence of ovoid or elongated procercarial embryos within the body of cercariae-containing rediae, even if these germinal masses were in small numbers. In contrast, in all experiments carried out by our team on French *G. truncatula* infected with local miracidia of *F. hepatica*, morulae and procercarial embryos have always disappeared from the redia body just when this larva contained several long-tailed cercariae [13].

Compared to the number of free cercariae noted in the French group (Figure 3), very small numbers of these larvae were counted in the body of snails dissected from day 42 up to day 112. This finding is more difficult to comment. Indeed, it is commonly admitted that *F. hepatica* cercariae exited from the body of their parent rediae and remained in large numbers within the body of snails. During this period, free cercariae accumulated glycogen and fatty acids before their shedding from the snail [5, 6]. These low numbers of free cercariae in the Argentinean group might be due to the two-week interval used in sample collections but this explanation cannot be retained because all infected snails from the Argentinean group only harboured a few free cercariae, whatever snail dissection (contrary to the French group which contained higher numbers of free cercariae from day 42 p.e. to day 84 (Figure 3). In our opinion, the most valid hypothesis is to admit that the accumulation period of these larvae within the snail body would be shorter than in the French group. This assumption can be sustained by the continuous shedding of cercariae over time that our team had noted in previous experiments on French *G. truncatula* infected with Argentinean *F. hepatica* miracidia [15, 16] because shedding waves [1, 3], during which most free cercariae were released from the body of snails, were scarce or did not occur in the Argentinean group. If the above hypothesis is considered valid, this raises once again the question of whether glycogen and fatty acid accumulation within the cercarial body would be sufficient to support a long period of encystment when this larva turns into a metacercaria and settles on a plant.

Different abiotic or biotic factors, reviewed by Rondelaud et al. [12, 13], had an effect on the development of *F. hepatica* redial generations and corresponding cercarial production, generally by limiting the numbers of second, third or fourth generation rediae. The main factor responsible for above-mentioned changes in the Argentinean group cannot be, at least in this case, an adaptation of the parasite to high altitude conditions existing in South America, as proposed by Mas-Coma et al. [7] to explain their results found in experimental infections of Bolivian lymnaeids with sympatric miracidia of *F. hepatica*. Indeed, the Argentinean isolate of miracidia used in the present study came from cattle living in plain lands (pampas), subjected to a temperate climate. Another more likely hypothesis may be that South American flukes would have followed a divergent evolutionary way after the introduction of mainly European infected ruminants in South America from the 15th century [8].

In conclusion, the changes in the redial development and cercarial production of Argentinean *F. hepatica* might explain the high cercarial production reported by Sanabria et al. [15, 16] in experimental infections of *G. truncatula* with this miracidial isolate. However, one may wonder if these changes are perennial by affecting the different successive generations of *G. truncatula* over time, or if they will disappear after several generations of snails, as that reported by Belfaiza et al. [2]. According to these authors, all first generation rediae gave birth to daughter rediae belonging to the second generation in French *G. truncatula* infected with a Moroccan isolate of *F. hepatica* miracidia. But, in the F1 and F2 generations of snails infected with the same isolate, there was a progressive return to a normal development of rediae with most second generation rediae produced by the first mother redia. Another way of research would be to study the characteristics of these isolates and to specify their major differences by molecular biology in order to determine if South American flukes have probably followed a divergent evolutionary way because of repeated introductions of *Bos taurus* herds into Latin America since the 15th century [8].
